# The impact of genetic testing for Crohn's disease, risk magnitude and graphical format on motivation to stop smoking: an experimental analogue study

**DOI:** 10.1111/j.1399-0004.2008.00964.x

**Published:** 2008-04

**Authors:** AJ Wright, C Takeichi, SCL Whitwell, M Hankins, TM Marteau

**Affiliations:** a Health Psychology, Department of Psychology, King's College LondonLondon, UK; b Department of Primary Care and Public Health, Brighton and Sussex Medical SchoolBrighton, UK; e-mail: barbarab@mail.nih.gov

**Keywords:** communication, Crohn's disease, genetic testing, motivation, risk, smoking cessation

## Abstract

Genetic tests may motivate risk-reducing behaviour more than other types of tests because they generate higher risk magnitudes and because their results have high personal relevance. To date, trial designs have not allowed the disentangling of the effects of these two factors. This analogue study examines the independent impacts of risk magnitude and provenance, and of risk display type, on motivation to quit smoking. A total of 180 smokers were randomly allocated to one of the 18 Crohn's disease risk vignettes in a 3 (risk provenance: family history. genetic test mutation positive. genetic test mutation negative) × 3 (risk magnitude: 3%, 6%, 50%) × 2 (display: grouped or dispersed icons) design. The 50% group had significantly higher intentions to quit than the 3% group. A significant risk provenance × magnitude interaction showed that participants in 50% or 6% groups were equally motivated, regardless of risk provenance, while participants in the 3% group had higher intentions associated with a mutation negative result than with a result based on family history alone. Grouped icon displays were more motivating than the dispersed icons. Using genetic tests to estimate risks of common complex conditions may not motivate behaviour change beyond the impact of the numerical risk estimates derived from such tests.

There are high expectations regarding the potential for DNA-based risk information to motivate health-related behaviour change more strongly than other types of risk information (1, 2). Two factors underpin these expectations. Firstly, DNA-based risk assessment procedures may generate a wider range of risk magnitudes than non-DNA-based risk assessments. For example, DNA testing can identify the individuals homozygous for a risk-conferring mutation who have a considerably increased likelihood of developing the condition in question relative to an average member of the population. These higher risk magnitudes generated by DNA-based risk assessments may increase perceived vulnerability to the health condition and thus motivate risk-reducing behaviour change better than do lower magnitude risk estimates generated by non-DNA-based risk assessments. This prediction is consistent with the evidence that providing disease risk estimates has a medium-sized effect on risk perceptions, which in turn have a small effect upon risk-reducing behaviour (3). Evidence from several theories of health behaviour (4–6) suggests that risk perceptions act in combination with other constructs to motivate behaviour change. Moreover, perceived vulnerability to the adverse effects of smoking is a factor influencing intentions to quit. For example, 69% of British smokers who wanted to quit said that this was due to their wish for better health (7).

The second reason why DNA-based risk information may better motivate behaviour change is that genetic test results may be viewed as more personally relevant than risk estimates based on family history but not incorporating genetic test results. Theories of attitude change predict that the greater the perceived personal relevance of information, the greater its impact (8). Consistent with this, diagnoses incorporating genetic test results can be perceived as more accurate than similar diagnoses made without the use of genetic tests and so may better motivate behaviour change (9).

Within the context of providing DNA-based risk estimates, individuals may react differently to mutation-positive and mutation-negative results. Genetic risks are viewed as less controllable than non-genetic risks (10). Given that lay people's representations of mutation-negative results may be that they ‘do not have the gene’ for the condition, not possessing a mutation may be interpreted as evidence that a personally relevant risk is controllable through behaviour change. In contrast, possessing the mutation may suggest that the risk is less controllable, so potentially demotivating behaviour change (11).

Few studies have explored whether DNA-based risk information does have greater motivational impact than numerical risk estimates derived from other sources and, if so, which of the two distinguishing features of genetic tests account for this. The small number of clinical studies conducted in this area suggest that the disclosure of genotypes indicating increased risk of disease is sometimes (12, 13), but not always (14, 15), associated with increased motivation for behaviour change. The current study aims to assess the extent to which any motivating impact of DNA-based risk information is due to the risk magnitudes generated by genetic tests or due to the higher perceived relevance of such information.

It is important to clarify the independent effects of the risk magnitudes generated by, and the greater perceived relevance of, genetic tests. This information will permit clinicians to forecast whether newly identified risk-conferring gene variants could form the basis of risk-reducing behaviour change interventions. If motivation is driven solely by risk magnitude, then gene variants that confer only small increases in the likelihood of developing a health problem are unlikely to motivate behaviour change. In contrast, if risk provenance, which affects perceived relevance of the information, contributes to motivation, then even risk assessments involving gene variants that confer small increases in risk magnitude may increase motivation relative to non-DNA-based risk assessments, and so have clinical utility.

Experimental analogue studies allow the effects of risk magnitude and risk provenance to be manipulated independently. Participants are asked to imagine themselves in a situation and to respond as if they had experienced the events described. Such studies have good internal validity and sufficient external validity to merit using the results to inform the design of clinically based studies (16–19). Several analogue studies have compared the motivational effects of genetic and non-genetic risk information in association with different risk magnitudes. Two studies (20, 21) found that risk magnitude, but not risk provenance, influenced intentions for risk-reducing behaviour. The other study (22) found evidence supportive of effects of both risk provenance and risk magnitude. However, the lack of significant differences in intentions between the low-risk, mutation-negative and the higher risk, non-DNA group in this study makes its findings difficult to interpret. Therefore, further evidence is needed to clarify the independent and interactive effects of risk magnitude and risk provenance on intentions for risk-reducing behaviour. Moreover, the potentially contrasting effects of mutation-positive and mutation-negative results have yet to be examined in a fully multifactorial risk provenance × risk magnitude design.

When trying to maximize the motivational impact of genetic risk information, a key question concerns how to present the risk estimates. Graphic formats can facilitate risk communication. Based on a recent review of such formats (23), it was decided to employ icon arrays to communicate probabilistic information. Icon arrays have the desirable property that, when icons are arranged as a group, the ability to estimate what proportion object A fills of the larger object B appears to be automatic (24), facilitating the communication of probabilities. An alternative arrangement involves randomly dispersing ‘affected’ icons throughout the array. Such dispersed displays may help convey the randomness inherent in probabilistic risk information, increasing perceived vulnerability to the adverse outcome and so motivating risk-reducing behaviour (23, 25). However, dispersed displays may cause difficulties estimating the proportion affected (26). The resultant comprehension problems may reduce the risk information's motivational impact. Therefore, the optimal display type to motivate behaviour change when providing genetic risk information remains to be determined.

The study focuses on communicating information about the risk of Crohn's disease, a chronic inflammatory disease of the digestive tract that can severely affect quality of life. Crohn's disease affects about 1 in 1000 people, runs in families and is more common in smokers. The average risk for smokers who have a sibling with Crohn's disease is 4.6%. The gene CARD15 has been identified as a susceptibility gene for Crohn's disease (27–30). The risk varies depending on the presence of a particular mutation that increases the likelihood of developing the disease. For smokers who have a sibling with Crohn's disease, the risk is about 35% when they are homozygous for CARD15, 8% when they are heterozygous for CARD15 and 5% when they have no mutation in the gene (31). We are planning a clinical trial (registered with Current Controlled Trials, ISRCTN 21633644) examining whether risk assessments for Crohn's disease including, or not including, the results of genetic tests influence motivation to quit. Other trials thus far have focused on whether communicating genetic risks of lung cancer motivates quitting (12–14).

The results of the current analogue study will provide evidence concerning which aspect(s) of genetic risk information most strongly motivate behaviour change. This information may help clinicians to anticipate contexts in which genetic risk information is more or less likely to succeed in motivating behaviour change. Thus, the present study aims to assess the independent effects of risk magnitude and risk provenance (genetic test, mutation positive; genetic test, mutation negative; based on family history alone) on intentions to stop smoking, and to determine how best to display this risk information to motivate behaviour change.

## Hypotheses

1. Genetic risk information generates greater intentions to stop smoking than risk information based only on family history.
2. Higher magnitudes of risk generate greater intentions to stop smoking.
3. Intentions to quit will differ between participants who view grouped and dispersed icon displays.

## Materials and methods

### Design

Between subjects, 3 (risk magnitude: 3%, 6%, 50%) × 3 (risk provenance: genetic test, mutation positive; genetic test, mutation negative; family history only) × 2(icon array: grouped or dispersed) experiment using vignettes.

### Participants

A total of 75 men and 105 women were recruited from the general population using a research agency (*n* = 140) and from staff and students of two universities (*n* = 40) (Table 1). There were 10 participants in each experimental group.

**Table 1:** Demographic characteristics and smoking status of the 180 study participants

Variable	Categories	*n*(%)
Gender	Male	75 (41.7)
	Female	105 (58.3)
Highest educational qualification	No formal qualifications	12 (6.7)
	GCSEs/O levelsa	39 (21.7)
	A levels/further educationb	43 (23.9)
	University degree	75 (41.7)
	Other qualifications	11 (6.1)
Time to first cigarette after waking	Within 5 min	25 (13.9)
	6–30 min	75 (41.7)
	31–60 min	37 (20.6)
	After 60 min	43 (23.9)
Number of cigarettes per day	1–5	30 (16.7)
	6–10	43 (23.9)
	11–20	63 (35.0)
	21–30	34 (18.9)
	31 or more	10 (5.6)

a British public examinations traditionally taken at age 16.

b British public examinations traditionally taken at age 18.

Participants smoked more than one cigarette per day and had not been diagnosed with Crohn's disease. Given the three substantive hypotheses outlined above, this sample size was estimated to be sufficient to detect a medium size main effect of each factor (*f* = 0.25) with α = 0.05 and power of 0.8. This sample size is also sufficient for subsequent pairwise comparisons with an adjusted α = 0.01.

### Measures

Intentions to stop smoking were assessed using two items (*r* = 0.89): ‘Do you intend to stop smoking in the next 4 weeks’, rated from 1:‘definitely not’ to 7: ‘definitely do’, and ‘How likely is it that you will stop smoking in the next four weeks?’ rated from 1:‘very unlikely’ to 7:‘very likely’. Perceived susceptibility to Crohn's disease was assessed with: ‘If you continue to smoke, how likely do you think it is that you will develop Crohn's disease’, rated from 1: ‘not at all’– 7:‘extremely likely’.

### Demographics and smoking behaviour

Participants began the questionnaire by giving their age, gender and educational qualifications. Nicotine dependence was assessed using the Heaviness of Smoking Index (32).

### Vignettes

**Fig. 1:**
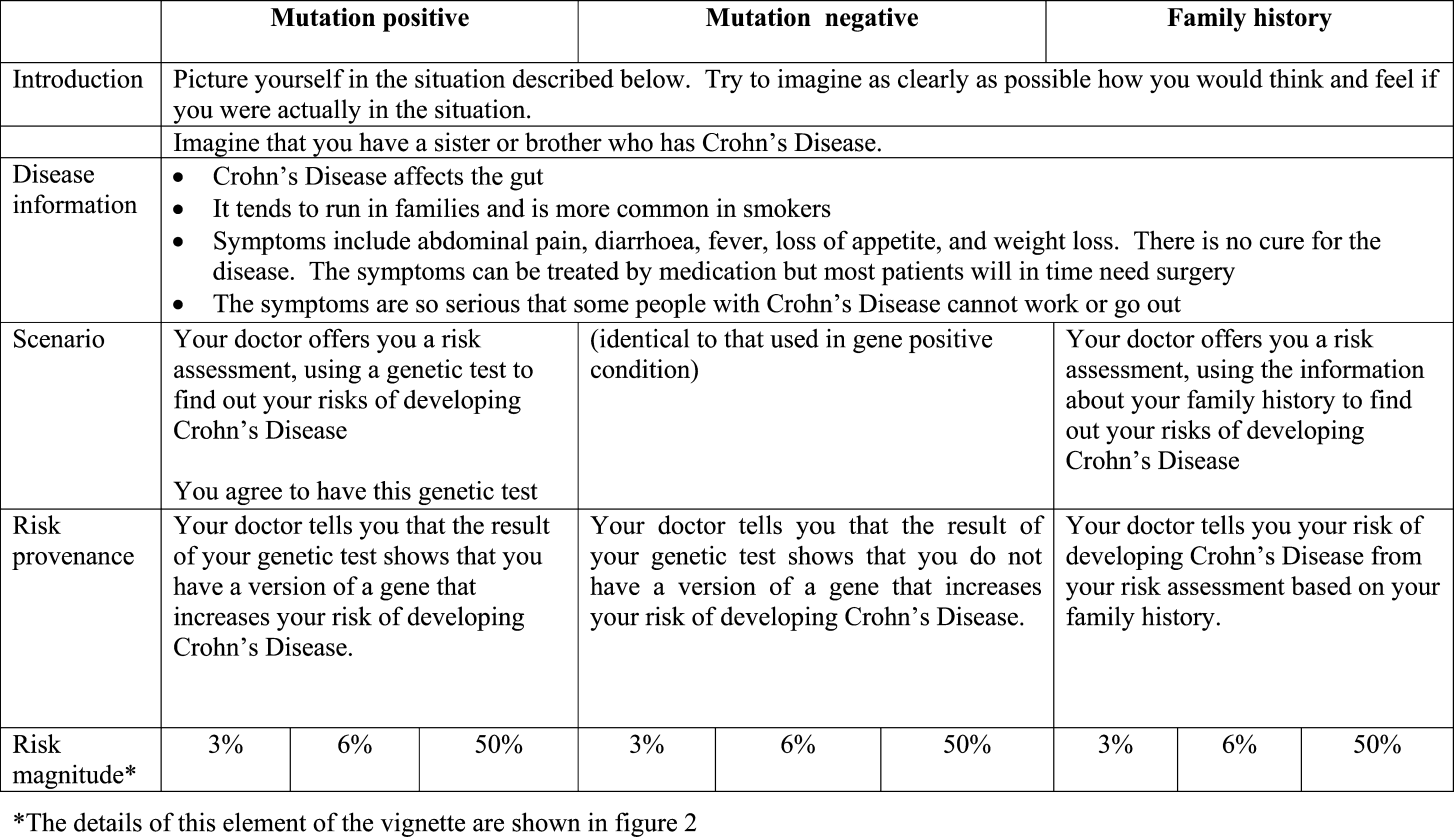
The vignettes used in the study.

**Fig. 2:**
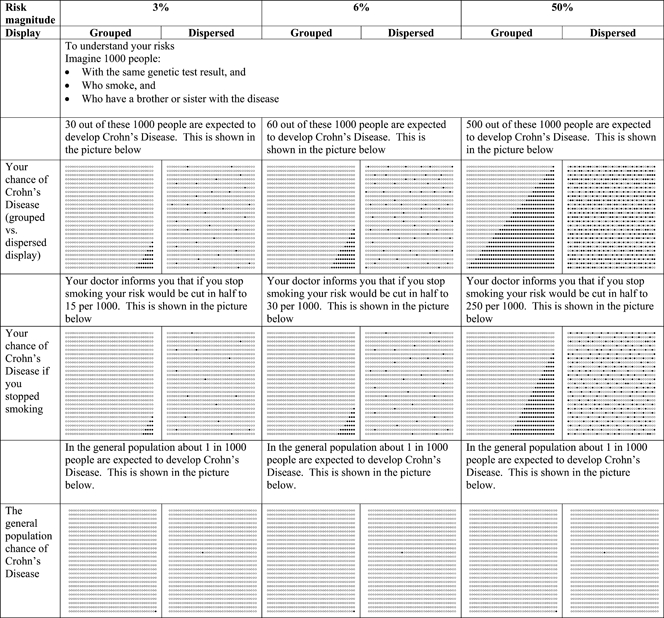
Details of how the different magnitude risk estimates were communicated.

Each vignette asked participants to imagine that they had a sibling with Crohn's disease, and provided information about the condition. Participants imagined undergoing a risk assessment to discover their own risk of developing Crohn's disease. They were then presented with their hypothetical risk assessment results and informed that by stopping smoking their risk would be halved. It should be noted that the risk magnitudes provided to participants are different to those cited in the *Introduction*. The figures used in the vignettes were believed, at the time the study was conducted, to accurately reflect smokers’ probability of developing Crohn's disease in the presence or absence of the CARD15 mutation. However, the risk magnitudes associated with the interaction between CARD15 and smoking behaviour have since been amended by the epidemiologists with whom we are collaborating, subsequent to further data collection (31). The full vignettes, including the graphical displays, are shown in Figs 1 and 2.

### Statistical analysis

The effects on intentions of risk provenance, risk magnitude, display type and their interactions were tested using anova. Simple main effects analysis was used to determine the form of any significant interactions.

### Procedure

King's College London Research Ethics Committee approved the study. Participants were recruited using the e-mail lists of two universities and a market research agency's Internet panel. Panel members complete surveys in return for ‘points’, which are exchangeable for various consumer goods at the agency's partner website. Participants were provided with 10 ‘points’ (approximate monetary value £1). Internet panel members were e-mailed a URL that linked to the vignettes and questionnaire online. University staff and students were e-mailed the study materials and questionnaire.

## Results

No differences were found between the 18 randomized groups on any of the demographic or smoking variables and so they were not used as covariates in the analysis.

### Effects on perceived susceptibility

Perceived susceptibility scores according to experimental group are shown in Table 2, while Table 3 provides the results of anova.

**Table 2:** Perceived susceptibility to Crohn's disease, according to risk provenance, risk magnitude and display

Risk provenance	Risk magnitude (%)	Display type			
		
		Dispersed		Grouped	
			
		Mean	SD	Mean	SD
Mutation positive	3	3.80	1.23	4.60	1.17
	6	4.50	1.51	3.50	1.58
	50	3.80	1.14	4.10	1.66
Mutation negative	3	3.60	1.43	4.50	1.08
	6	3.60	1.17	3.50	0.97
	50	4.20	1.55	4.10	1.10
Family history only	3	3.70	1.64	2.90	1.20
	6	4.20	1.69	4.60	1.58
	50	4.70	1.42	4.80	1.14

**Table 3:** Results of anova examining effects of risk magnitude, risk provenance and display type on perceived susceptibility to Crohn's disease

Effect	d.f.	F	p	Partial η^2^
Risk magnitude	2	1.583	0.209	0.019
Risk provenance	2	0.440	0.645	0.005
Display type	1	0.074	0.785	0.000
Risk magnitude × risk provenance	4	2.922	0.023	0.067
Risk magnitude × display type	2	0.583	0.559	0.007
Risk provenance × display type	2	0.226	0.798	0.003
Risk magnitude × risk provenance × display type	4	1.833	0.125	0.043

The main effect of risk magnitude on perceived susceptibility was not significant. However, the means were in the predicted direction, with those in the 3% group reporting lower susceptibility (mean = 3.85) than the 6% group (mean = 3.98), who in turn reported lower susceptibility than the 50% group (mean = 4.28). There was also a significant risk magnitude × risk provenance interaction. Simple main effects analysis using a Sidak adjustment for multiple comparisons found that perceived susceptibility was affected by risk magnitude in the family history only condition [simple main effect F(2,162) = 6.13, p = 0.003], but not in either of the genetic testing conditions. Pairwise comparisons revealed that, within the family history condition, the 3% group perceived significantly lower susceptibility to Crohn's disease than did the 6% or 50% groups, while the latter two did not differ in their perceptions of susceptibility.

### Effects on intentions to quit

Mean intentions according to experimental group are shown in Table 4, while Table 5 shows the results of the anova.

**Table 4:** Intentions to quit smoking, according to risk provenance, risk magnitude and display

Risk provenance	Risk magnitude (%)	Display type			
		
		Dispersed		Grouped	
			
		Mean	SD	Mean	SD
Mutation positive	3	2.50	1.27	3.20	1.93
	6	2.85	1.55	3.00	1.56
	50	3.75	2.29	4.30	1.55
Mutation negative	3	2.75	1.34	4.25	1.16
	6	3.10	2.07	2.25	1.34
	50	3.30	1.99	3.90	1.68
Family history only	3	2.15	1.49	2.15	1.87
	6	2.80	1.44	4.60	1.70
	50	2.90	1.56	3.85	1.84

**Table 5:** Results of anova examining effects of risk magnitude, risk provenance and display type on intentions to quit smoking

Effect	d.f.	F	p	Partial η^22^
Risk magnitude	2	3.890	0.022	0.046
Risk provenance	2	0.252	0.778	0.003
Display type	1	5.798	0.017	0.035
Risk magnitude × risk provenance	4	2.918	0.023	0.067
Risk magnitude × display type	2	0.221	0.802	0.003
Risk provenance × display type	2	0.407	0.666	0.005
Risk magnitude × risk provenance × display type	4	1.946	0.105	0.046

The main effect of risk provenance was not significant, but there was a significant main effect of risk magnitude on intentions to quit. Sidak-adjusted pairwise comparisons showed that participants who received a 50% risk estimate were significantly more motivated than participants who received a 3% risk estimate. The 6% group fell between these two extremes, not differing significantly from either.

This effect of risk magnitude on intentions was modified by a significant risk magnitude × risk provenance interaction. This took the form that, for participants receiving 50% or 6% risk estimates, risk provenance made no difference to intentions to quit. However, participants in the 3% risk estimate group had stronger intentions when the risk estimate was based on a mutation negative result compared with when it was based on family history alone [simple main effect of risk provenance in the 3% group: F(2,162) = 3.26, p = 0.04]. Finally, the significant main effect of display type was due to participants who viewed a grouped display having higher intentions than participants who viewed a dispersed display.

## Discussion

This study examined the effects of risk estimate provenance, risk magnitude and display type on motivation to quit smoking. The greater the magnitude of the risk estimates the stronger were the participants’ intentions to adopt risk-reducing behaviour. Mean perceived susceptibility to Crohn's disease was higher in the higher risk magnitude groups, suggesting that risk magnitude influenced susceptibility and so intentions. However, this interpretation must be considered tentative, as although the pattern of mean susceptibility scores was as predicted, the size of the differences was not sufficient to be statistically significant.

Only when the magnitude of the risk estimate was lowest (3%) did risk provenance make a difference to intentions. Participants in the 3% group for whom the estimate was based on a mutation-negative result were more motivated than participants receiving an estimate of identical magnitude based on family history alone. Intentions for those in the mutation-positive group were intermediate between these two extremes. One possible explanation for this finding is that genetic test results were perceived as more relevant and so motivating than were risk estimates based on family history alone. However, mutation-positive results may also have been interpreted as suggesting that the likelihood of developing Crohn's disease was less controllable, resulting in this group having lower intentions to quit than the mutation-negative group. Given this interaction was not predicted, replication is required to ensure it is a robust finding.

The significant main effect of risk magnitude on intentions echoes the findings of previous vignette studies (20–22). However, the finding of a significant risk magnitude × provenance interaction differs from that of the only other vignette study to test this interaction (20). This discrepancy could result from the differing values of the risk magnitudes used in two studies, or from the current study presenting both relative and absolute risk estimates, whereas the earlier study communicated only relative risks.

Presenting the probabilistic information using grouped icons led to greater intentions to quit than when the same information was presented using dispersed icon displays. This finding accords with the other work, which suggests that grouped displays make it easier for participants to appreciate the relative magnitudes of risks and so may more strongly motivate risk-reducing behaviour (23), and with the suggestion that dispersed displays may cause comprehension difficulties and so reduce motivation. It appears that presenting risk estimates associated with genetic risk information using grouped icon displays may maximize any motivational effects.

## Strengths, limitations and suggestions for further research

This study's major strength is that it allowed the motivational effects of risk provenance to be disentangled from those of risk magnitude, and that it includes both mutation-positive and mutation-negative genetic testing conditions. The study also benefits from using a sample reasonably representative of British smokers (33). Experimental vignettes cannot completely reflect the nature of complex encounters between health professionals communicating risk information and their patients. However, analogue methods permitted the separation of two key factors that would not be possible to achieve in a clinical context. One further limitation is that there was a small error in the second page of information seen by the family-history-based risk estimate groups. On the first page of information, although genetic testing was not mentioned, on the second page participants were asked to ‘Imagine 1000 people, with the same genetic test result, and who smoke, and who have a brother or sister with the disease’. This mention of a genetic test result in what was intended to be a family-history-only condition may have obscured the motivational impact of risk provenance. However, the effect size for the main effect of risk provenance was less than one-tenth that for display type or magnitude. Therefore, it seems unlikely that risk provenance would have significantly influenced intentions even if this error had not been made.

Clinicians and researchers investigating whether genetic risk information can motivate risk-reducing behaviour change should include further manipulation checks on participants’ beliefs to facilitate understanding of the processes causing any observed differences in motivation for, and practice of, risk-reducing behaviours. Such measures should include participants’ perceptions of the precision and personal relevance of the risk estimate, which we predict will be greater in conditions where risk estimates include a genetic test result than in conditions where risk estimates are not based on a genetic test result. Future research would also benefit from deeper exploration of participants’ perceptions of the risk information, including whether high-magnitude risk estimates are viewed as credible and how well participants comprehend the information.

## Conclusions

Higher risk magnitudes better motivate behaviour change, regardless of whether they are derived from genetic tests or not. Risks presented using grouped, as opposed to dispersed, displays are also more motivating. Although the motivational impact of genetic testing may be most evident following the presentation of risks of low magnitude, the results of this study are compatible with the hypothesis that using genetic tests to estimate risks of common complex conditions will not motivate behaviour change beyond the motivating impact of the risk magnitudes derived from such tests.
